# Plasma Adiponectin Levels Correlate Positively with an Increasing Number of Components of Frailty in Male Elders

**DOI:** 10.1371/journal.pone.0056250

**Published:** 2013-02-13

**Authors:** Jaw-Shiun Tsai, Chih-Hsun Wu, Su-Chiu Chen, Kuo-Chin Huang, Chin-Ying Chen, Ching-I Chang, Lee-Ming Chuang, Ching-Yu Chen

**Affiliations:** 1 Department of Family Medicine, College of Medicine, National Taiwan University, Taipei, Taiwan; 2 Department of Internal Medicine, College of Medicine, National Taiwan University, Taipei, Taiwan; 3 Graduate Institute of Clinical Medicine, College of Medicine, National Taiwan University, Taipei, Taiwan; 4 Department of Psychology, National Taiwan University, Taipei, Taiwan; 5 Department of Health Care Management, National Taipei University of Nursing and Health Sciences, Taipei, Taiwan; 6 Division of Geriatrics and Gerontology, Institute of Population Health Science, National Health Research Institutes, Ju-Nan, Taiwan; Wageningen University, The Netherlands

## Abstract

**Objective:**

Frailty is an important geriatric syndrome. Adiponectin is an important adipokine that regulates energy homeostasis. The aim of this study is to investigate the relationship between plasma adiponectin levels and frailty in elders.

**Methods:**

The demographic data, body weight, metabolic and inflammatory parameters, including plasma glucose, total cholesterol, triglyceride, tumor necrosis factor alpha (TNF-α), c-reactive protein (CRP) and adiponectin levels, were assessed. The frailty score was assessed using the Fried Frailty Index (FFI).

**Results:**

The mean (SD) age of the 168 participants [83 (49.4%) men and 85 (50.6%) women] was 76.86 (6.10) years. Judged by the FFI score, 42 (25%) elders were robust, 92 (54.7%) were pre-frail, and 34 (20.3%) were frail. The mean body mass index was 25.19 (3.42) kg/m^2^. The log-transformed mean (SD) plasma adiponectin (µg/mL) level was 1.00 (0.26). The log-transformed mean plasma adiponectin (µg/mL) levels were 0.93 (0.23) in the robust elders, 1.00 (0.27) in the pre-frail elders, and 1.10 (0.22) in the frail elders, and the differences between these values were statistically significant (*p*  = 0.012). Further analysis showed that plasma adiponectin levels rose progressively with an increasing number of components of frailty in all participants as a whole (*p* for trend  = 0.024) and males (*p* for trend  = 0.037), but not in females (*p* for trend  = 0.223).

**Conclusion:**

Plasma adiponectin levels correlate positively with an increasing number of components of frailty in male elders. The difference between the sexes suggests that certain sex-specific mechanisms may exist to affect the association between adiponectin levels and frailty.

## Introduction

Frailty is a geriatric syndrome associated with decreased physiologic reserve, functional decline, and increased vulnerability to stressors, which lead to disability and mortality [1]. The mechanism of frailty is multifactorial. Inadequate nutrition and impairment and dysfunction of the endocrine and immune systems are involved in the development of frailty [2]. Fried’s concept of frailty postulates that frailty is initiated by the accumulation of factors such as lack of physical exercise, inadequate nutrition, injuries, disease, and drugs [3]. These factors may lead to chronic undernutrition, resulting in loss of bone and skeletal muscle mass and an increase in the degree of effort required for a given exercise intensity. Elders are more likely to avoid exercise as they believe that more effort is required. These changes may result in a significant decrease in resting energy metabolism and total energy expenditure [4].

Adiponectin is an important adipokine which possesses insulin sensitizing, anti-atherosclerotic and anti-inflammatory properties [5]. Studies have shown a negative correlation between the circulating levels of adiponectin and obesity, insulin resistance, coronary artery disease, and dyslipidemia [6], [7]. High levels of circulating adiponectin have been associated with a reduced risk of cardiovascular disease (CVD) in some population-based studies [8], [9]. In addition, adiponectin has a potential role in the central regulation of energy intake and expenditure [10]. Thus, circulating adiponectin may play a potential role in geriatric frailty.

A number of epidemiological studies on adiponectin have been conducted in aged populations. Adiponectin levels were significantly associated with high-density lipoprotein cholesterol (HDL-C) concentrations in postmenopausal women, which suggested that high adiponectin levels may have a protective effect against atherosclerosis, when the HDL-C concentrations are high [11]. The inverse relationship between regional fat depots and the risk of the metabolic syndrome may be partially mediated by the adiponectin levels and the inflammatory status of middle-aged and older Chinese men and women [12]. Plasma concentrations of adiponectin were also high in middle-aged and older Chinese people with high levels of total physical activity [13]. However, adiponectin levels are known to increase with age [14], and this increase occurs in spite of the increase in visceral fat and insulin resistance that occurs with normal aging [15]. Furthermore, raised adiponectin levels are associated with increased rather than decreased risk of CVD and mortality in older subjects [16]. A study also showed that high levels of adiponectin predict mortality, particularly in patients with prevalent CVD [17]. In addition, a longitudinal study showed that circulating adiponectin levels increase over time in long-lived adults and are associated with greater physical disability and mortality [18]. These conflicting findings suggested a possibility that adiponectin may have different prognostic implications in older subjects [16].

Although a recent study showed lower fasting levels of adiponectin in frail women, the result was not statistically significant [19]. Thus, the association between plasma adiponectin levels and frailty in the older population warrants more investigation in detail. We aimed to investigate the relationship between plasma adiponectin levels and geriatric frailty in both men and women. We hypothesized that plasma adiponectin levels increase with frailty in elderly.

## Methods

### Ethics Statement

The study protocol was approved by the Ethics Committee of the National Taiwan University Hospital (registration number: 200701017R), and written informed consent was obtained from all participants before their inclusion in the study. The items of the consent form include aim, inclusion and exclusion criteria, procedures, harm and benefit, medical care, privacy and right, and withdrawal. All procedures were in accordance with the Helsinki Declaration. In addition, all potential participants who declined to participate or otherwise did not participate were still in care of their family physicians and were not disadvantaged in any other way by not participating in the study.

### Subjects

From January 2007 to June 2008, the older patients who were aged 65 years and over and were followed up for chronic diseases with their family physicians in a hospital-based program were recruited for a structured comprehensive geriatric assessment if any of the following inclusion criteria was met. These criteria included: (1) functional decline (as measured by new disabilities of activity of daily living or instrumental activity daily living); (2) clinical indication of depression or dementia; (3) mobility impairment; (4) fall in recent one year; (5) weight loss more than 5% per year; (6) multiple comorbidities (≥5 diseases); (7) polypharmacy (≥8 classes of drugs per day); (8) multiple specialty physician visits in recent 6 months (≥3 different specialties with ≥2 visits for each specialty); (9) hospitalization in the past 1 year (≥1); (10) frequent emergency room visits in the past year (≥2); and (11) age above 80 years. The exclusion criteria were patients who were bedridden or residing in a nursing home, patients with an expected life expectancy of less than 6 months, and patients with a severe hearing or communication disorder.

### Data Collection

The experienced study nurses collected the data with a structured questionnaire, which included history on demographics, diseases, smoking and drinking habits, current medication, geriatric syndromes, blood pressure level, and body mass index (BMI). The Frailty Index was assessed by modified Fried’s criteria [3]; “weight loss” was defined as self-reported, unintentional weight loss of more than 3 kg (instead of 5 kg, adjusted in proportion to the Chinese body build) or greater than 5% of the body weight in the previous year. “Exhaustion” was indicated if the participants responded with “a moderate amount of the time” or “most of the time” to either of the following 2 statements: “I felt everything I did was an effort” or “I could not get going.” The statements were obtained from the Center for Epidemiological Studies-Depression Scale [20]. “Low physical activity” was defined by sex-specific, low weekly energy expenditure measured using the Taiwan International Physical Activity Questionnaire-Short Form (IPAQ-SF) [21] instead of the Minnesota Leisure Time Physical Activity Questionnaire [22]. “Slow walking speed” based on the time to walk for 5 meters was below certain sex- and height-specific cut-points [3]. “Weakness” was indicated when the maximal grip strength (kilograms) in the dominant hand (3 grips averaged), using a Jamar hand-held dynamometer was lesser than certain sex- and BMI-specific cut-points [3]. The subjects were classified as “robust,” “pre-frail,” or “frail” when 0, 1 or 2, or ≥3 components, respectively, screened positive [3].

### Biochemical Assays and Adiponectin Measurement

Blood samples for the measurement of the adiponectin levels, complete blood count, and biochemical analysis were obtained from the antecubital vein of the subjects after an 8-h fast. For the adiponectin study, the blood was then immediately centrifuged. The plasma samples obtained after centrifugation were then frozen at −70°C until analysis. Total plasma adiponectin levels were determined using a previously described radioimmunoassay method (Linco Research, Inc., St. Charles, MO) [23]. Plasma TNF-α levels were measured by the commercial enzyme-linked immunosorbent assay (ELISA) kits (Assaypro LLC, Saint Charles, Missouri, USA). Plasma CRP levels were measured by the latex agglutination test (Denka Seiken, Gosen, Niigata, Japan).

### Statistical Analyses

Descriptive statistical data were summarized as frequencies and percentages for categorical variables and means and standard deviations for other continuous variables. An analysis of variance (ANOVA) was used to examine significant differences in the adiponectin levels of the three frailty subgroups. A general linear model was used to test if there is a trend between frailty scores and adiponectin concentrations (log-transformed) after adjusted for age and BMI for both sexes and for all participators. A probability of less than 0.05 (*p*<0.05) was considered statistically significant. All data were analyzed by using SPSS 14.0 statistical software (SPSS Inc., Chicago, IL).

## Results

### Plasma Adiponectin Levels in the Study Subjects

There were 241 patients aged 65 years and over who met the inclusion criteria. Fifty-two patients declined to join the study and 189 patients completed the assessment and frailty screening. Among the 189 participants, 168 had their blood sample collected. Of the 168 elders, 83 (49.4%) were men. Their mean (SD) age was 76.86 (6.10) years. Fifty-one (30.4%) elders had quitted cigarette smoking and only nine (5.4%) smoked cigarettes. The leading comorbidities were hypertension (82.7%), hyperlipidemia (61.3%), diabetes mellitus (43.5%), and coronary artery disease (31.5%). Judged by the frailty scoring, 42 (25.0%) elders were robust, 92 were (54.7%) pre-frail, and 34 (20.3%) were frail (Table 1). Their medication data are summarized in Table 1. There was no significant difference in demographic variables except education between male and female subgroups. The mean (SD) BMI was 25.19 (3.42) kg/m^2^. The log-transformed mean (SD) plasma adiponectin (µg/mL), TNF-α (pg/mL) and CRP (nmol/L) levels were 1.00 (0.26), 1.51 (0.41), and 1.42 (0.29) respectively (Table 2). Other laboratory data results are summarized in Table 2. Plasma total cholesterol (*p*  = 0.001) and uric acid (*p*  = 0.022) concentrations were significantly different between male and female subgroups.

**Table 1:** Demographic data of study participants.

	Overall (n = 168)	Male (n = 83)	Female (n = 85)	
Variables	Statistics^a^	Statistics^a^	Statistics^a^	*t(p)/χ^2^(p)*
Age	76.86±6.10	77.71±5.87	76.04±6.24	1.79 (.075)
Education				
Less than 6 years	86 (51.2%)	31 (37.3%)	55 (64.7%)	17.99 (.000)
Junior High	23 (13.7%)	10 (12.0%)	13 (15.3%)	
Senior High	24 (14.3%)	16 (19.3%)	8 (9.4%)	
College or more	35 (20.8%)	26 (31.3%)	9 (10.6%)	
Smoking status				
Never	108 (64.3%)	25 (30.1%)	83 (97.6%)	N/A
Quitted	51 (30.4%)	50 (60.2%)	1 (1.2%)	
Smoking	9 (5.4%)	8 (9.6%)	1 (1.2%)	
Comorbidity				
Hypertension	139 (82.7%)	66 (79.5%)	73 (85.9%)	1.19 (.275)
Hyperlipidemia	103 (61.3%)	47 (56.6%)	56 (65.9%)	1.52 (.218)
Diabetes mellitus	73 (43.5%)	34 (41.0%)	39 (45.9%)	0.41 (.520)
Coronary artery disease	53 (31.5%)	27 (32.5%)	26 (30.6%)	0.07 (.787)
Stroke	45 (26.8%)	25 (30.1%)	20 (23.5%)	0.93 (.335)
Medication				
Aspirin	74 (44.0%)	39 (47.0%)	35 (41.2%)	0.57 (.448)
β-blockers	41 (24.4%)	21 (25.3%)	20 (23.5%)	0.07 (.789)
Calcium channel blockers	80 (47.6%)	38 (45.8%)	42 (49.4%)	0.22 (.638)
ACEIs or ARBs	94 (56.0%)	46 (55.4%)	48 (56.5%)	0.02 (.891)
Metformin	44 (26.2%)	18 (21.7%)	26 (30.6%)	1.72 (.190)
Sulfonylureas	53 (31.5%)	22 (26.5%)	31 (36.5%)	1.93 (.165)
Thiazolidinediones	13 (7.7%)	6 (7.2%)	7 (8.2%)	0.06 (.807)
Acarbose	5 (3.0%)	3 (3.6%)	2 (2.4%)	N/A
Repaglinide	4 (2.4%)	1 (1.2%)	3 (3.5%)	N/A
Statins	56 (33.3%)	22 (26.5%)	34 (40.0%)	3.44 (.064)
Frailty Score (Level)				
0 (Robust)	42 (25.0%)	22 (26.5%)	20 (23.5%)	N/A
1 (Pre-frail)	53 (31.5%)	30 (36.1%)	23 (27.1%)	
2 (Pre-frail)	39 (23.2%)	17 (20.5%)	22 (25.9%)	
3 (Frail)	24 (14.3%)	9 (10.8%)	15 (17.6%)	
4 (Frail)	9 (5.4%)	5 (6.0%)	4 (4.7%)	
5 (Frail)	1 (0.6%)	0 (0.0%)	1 (1.2%)	

Note. a: n (%) for categorical data, mean (SD) for continuous data.

N/A: Chi-square not available due to having cells with less than 5 cases.

No variable was adjusted for those models in this table.

ACEIs: angiotensin-converting enzyme inhibitors; ARBs: angiotensin II receptor blockers.

**Table 2:** Results of the physical examination and laboratory tests of the 168 elders.

	Overall		Male		Female		
	Mean	(SD)	Mean	(SD)	Mean	(SD)	*t(p)*
Physical examination							
Height (cm)	156.67	(8.32)	162.83	(6.37)	150.65	(4.89)	13.88 (.000)
Weight (kg)	61.94	(10.53)	66.65	(10.54)	57.33	(8.27)	6.38 (.000)
Body mass index (kg/m^2^)	25.19	(3.42)	25.12	(3.56)	25.25	(3.30)	−0.24 (.813)
Blood Pressure							
Systolic (mmHg)	131.48	(16.73)	132.55	(18.93)	130.42	(14.30)	0.82 (.411)
Diastolic (mmHg)	75.13	(8.62)	75.51	(8.56)	74.76	(8.71)	0.56 (.000)
Laboratory tests							
RBC (M/µL)	4.38	(0.58)	4.49	(0.65)	4.27	(0.47)	2.42 (.017)
Hb (g/dL)	12.98	(1.60)	13.37	(1.75)	12.58	(1.34)	3.05 (.003)
Platelet (K/µL)	223.01	(71.77)	205.92	(67.09)	240.11	(72.66)	−2.97 (.003)
WBC (K/µL)	6.54	(1.55)	6.51	(1.47)	6.56	(1.64)	−0.20 (.843)
Albumin (g/dL)	4.56	(0.33)	4.54	(0.42)	4.57	(0.23)	−0.39 (.697)
Glucose AC (mmol/L)	6.50	(1.92)	6.40	(1.97)	6.60	(1.89)	−0.65 (.519)
Total-Cholesterol (mmol/L)	4.89	(0.88)	4.64	(0.80)	5.11	(0.90)	−3.41 (.001)
Triglyceride (mmol/L)	1.69	(1.01)	1.56	(0.75)	1.81	(1.20)	−1.55 (.122)
AST (µkat/L)	0.41	(0.20)	0.43	(0.25)	0.39	(0.14)	1.01 (.312)
ALT (µkat/L)	0.37	(0.24)	0.39	(0.28)	0.34	(0.19)	1.45 (.148)
BUN (mmol/L)	0.75	(0.34)	0.83	(0.40)	0.67	(0.23)	2.95 (.003)
Creatinine (µmol/L)	110.12	(48.31)	129.77	(58.77)	91.23	(23.47)	5.29 (.000)
MDRD-simplify-GFR (mL/min/1.73 m^2^)	57.88	(16.37)	56.58	(17.55)	59.12	(15.16)	−0.96 (.338)
Uric acid (µmol/L)	385.81	(95.48)	403.84	(95.73)	367.28	(92.27)	2.32 (.022)
Log(TNF-α (pg/mL))	1.51	(0.41)	1.50	(0.41)	1.52	(0.40)	−0.30 (.762)
Log (CRP (nmol/L))	1.42	(0.29)	1.44	(0.29)	1.41	(0.30)	−0.50 (.619)
Log (Adiponectin (µg/mL))	1.00	(0.26)	0.97	(0.22)	1.03	(0.28)	−1.30 (.195)

MDRD: Modification of diet in renal disease.

MDRD-simplify-GFR (mL/min/1.73 m^2^) = 186 × [(CRE)^−1.154^] × [(age)^−0.203^] (if male).

MDRD-simplify-GFR (mL/min/1.73 m^2^) = 186 × [(CRE)^−1.154^] × [(age)^−0.203^] × 0.742 (if female).

No variable was adjusted for those models in this table.

### Plasma Adiponectin Levels in the Three Subgroups with Different Frailty Levels

**Figure 1:**
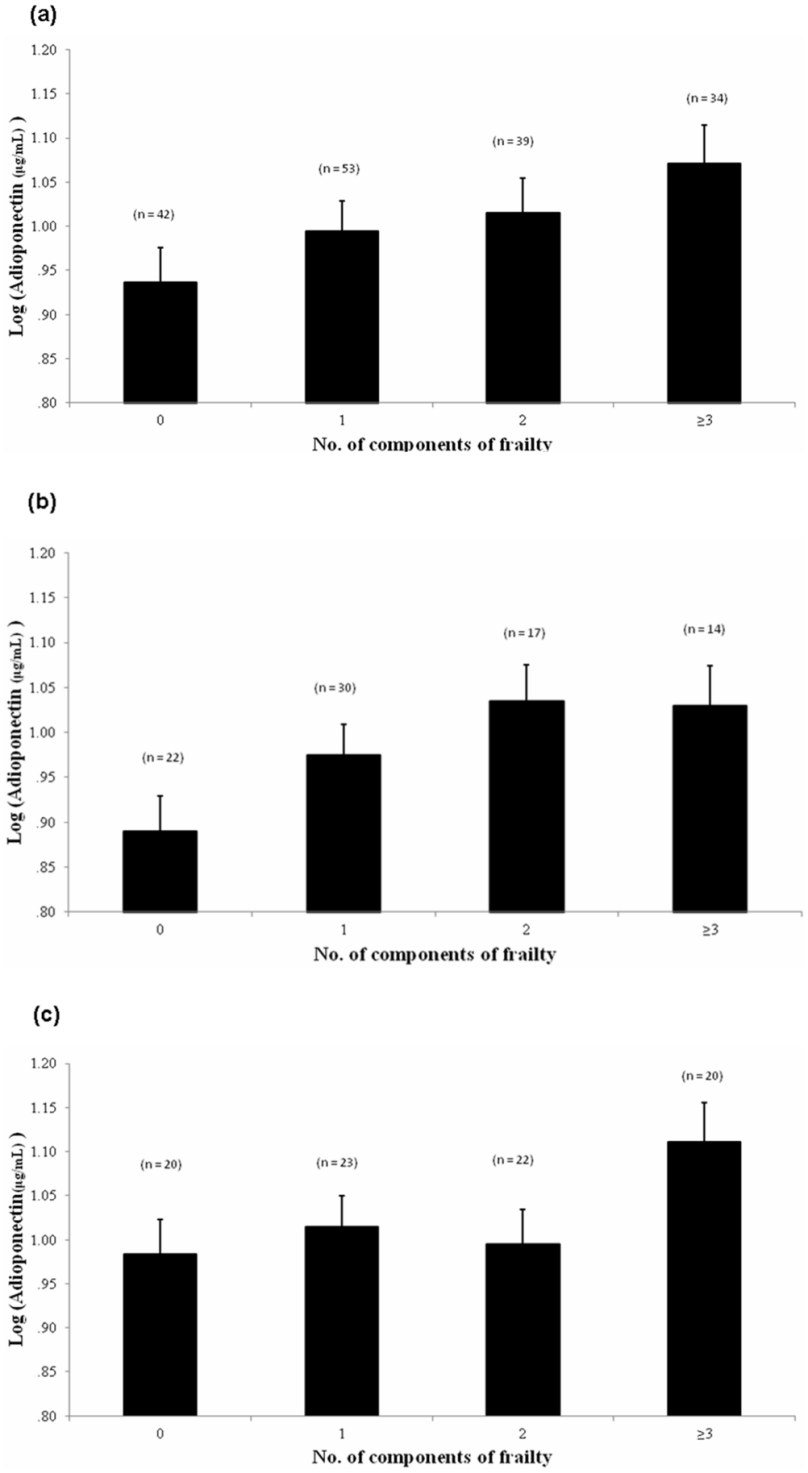
Adiponectin concentration (log-transformed) elevated progressively with increasing number of components of frailty while age and BMI were controlled. *p* for trend: (a) total: *p*  = 0.024, (b) males: *p*  = 0.037, (c) females: *p*  = 0.223.

Among the three subgroups, participants in the frail subgroup were significantly older than those in the other two subgroups. The log-transformed mean (SD) plasma adiponectin (µg/mL) levels in the robust, pre-frail, and frail subgroups were 0.93 (0.23), 1.00 (0.27), and 1.10 (0.22), respectively. The plasma adiponectin levels were significantly different among the three subgroups (*p*  = 0.012). The results of the post hoc tests showed that the people in the frail subgroup had higher plasma adiponectin levels than those in the robust subgroup. We further analyzed the effect of sex on the relationship between frailty severity and plasma adiponectin level. In both subgroups, plasma adiponectin levels were not associated with frailty severity. With regard to comorbidity and medication, a lower percentage of people in the robust subgroup had DM (23.8%) and stroke (11.9%) and used aspirin (33%). However, a higher percentage of people in the robust subgroup had hyperlipidemia (76.2%) and used statins (52.4%) (Table 3). The results of the hemogram and the biochemical analysis were not different among the three subgroups (data not shown). However, it was very interesting that plasma adiponectin levels rose progressively with an increasing number of components of frailty in all participants as a whole (*p* for trend  = 0.024) and males (*p* for trend  = 0.037), but not in females (*p* for trend  = 0.223) (Figure 1).

**Table 3:** Comparisons among elders with different frailty levels.

	Frailty			
	Robust	Pre-frail	Frail	
	Statistics^a^	Statistics^a^	Statistics^a^	*F(p)/χ^2^(p)*
Age	74.69±6.68	77.04±5.97	79.06±4.86	5.14 (.007)
Sex (female)	20 (47.6%)	45 (48.9%)	20 (58.8%)	1.17 (.556)
Body mass index (kg/m^2^)	25.04±3.32	25.52±3.55	24.46±3.15	1.26 (.287)
MDRD-simplify-GFR (mL/min/1.73 m^2^)	62.43±12.56	56.68±18.23	55.62±14.81	2.04 (.143)
Log (TNF-α (pg/mL))	1.55±0.38	1.50±0.41	1.47±0.43	0.36 (.699)
Log (CRP (nmol/L))	1.42±0.30	1.42±0.28	1.43±0.32	0.03 (.974)
Log (Adiponectin (µg/mL)):				
Overall	0.93±0.23	1.00±0.27	1.10±0.22	4.51 (.012)
Male	0.89±0.21	0.99±0.23	1.06±0.18	2.66 (.076)
Female	0.96±0.25	1.01±0.31	1.13±0.25	1.92 (.154)
Diseases				
Hypertension	38 (90.5%)	75 (81.5%)	26 (76.5%)	2.79 (.248)
Diabetes mellitus	10 (23.8%)	44 (47.8%)	19 (55.9%)	9.45 (.009)
Hyperlipidemia	32 (76.2%)	57 (62.0%)	14 (41.2%)	9.75 (.008)
Stroke	5 (11.9%)	25 (27.2%)	15 (44.1%)	9.96 (.007)
CAD	13 (31.0%)	33 (35.9%)	7 (20.6%)	2.69 (.260)
Medication				
Aspirin	14 (33.3%)	39 (42.4%)	21 (61.8%)	6.39 (.041)
β-blockers	12 (28.6%)	21 (22.8%)	8 (23.5%)	0.53 (.766)
CCBs	20 (47.6%)	47 (51.1%)	13 (38.2%)	1.64 (.440)
ACEIs or ARBs	24 (57.1%)	48 (52.2%)	22 (64.7%)	1.61 (.446)
Metformin	7 (16.7%)	24 (26.1%)	13 (38.2%)	4.52 (.104)
Sulfonylureas	9 (21.4%)	31 (33.7%)	13 (38.2%)	2.89 (.235)
Statins	22 (52.4%)	29 (31.5%)	5 (14.7%)	12.30 (.002)

CAD: coronary artery disease; CCBs: calcium channel blockers; ACEIs: angiotensin-converting enzyme inhibitors; ARBs: angiotensin II receptor blockers.

[Post hoc: Frail>Robust for AP and log (AP)].

No variable was adjusted for those models in this table.

## Discussion

This study was performed in 168 elderly subjects aged between 65 and 90 years. We identified that high plasma adiponectin levels are associated with frailty in elders. Furthermore, plasma adiponectin levels correlate positively with an increasing number of components of frailty in male elders. Our findings support the link between high adiponectin levels and mortality in the general older population [16], [18].

A recent study on 73 community-dwelling women, aged 84–95 years without a diagnosis of diabetes, showed fasting levels of adiponectin were lower in frail subjects, but not significant statistically [19]. There are two important differences in the study subjects between their study and ours. First, their study population did not have diabetes whereas 43% of our study subjects did. Second, their study population was limited in women only. However, both studies found no significant relationship between plasma adiponectin levels and frailty in females.

Geriatric frailty, which is usually concurrent with sarcopenia and aging, is associated with increased inflammatory activity and is reflected by increased levels of circulating TNF-α, interleukin (IL)-6, cytokine antagonists, and acute-phase proteins. Epidemiologic studies suggest that chronic low-grade inflammation during aging promotes an atherogenic profile and is related to age-associated disorders, such as Alzheimer’s disease, atherosclerosis, type 2 diabetes and enhances mortality risk. The development of frailty is characterized by the dysregulated production of proinflammatory cytokines [24]. Therefore, frailty is a critical condition in the elderly. It is noteworthy that plasma adiponectin levels are high in frail elders, which has anti-inflammatory, insulin sensitization, and anti-atherogenic properties [25]. However, plasma TNF-α and CPR levels were not elevated in the frail elders in our study. Thus, elevation of plasma adiponectin levels in the frail elders is not due to compensatory upregulation. A recent study has shown that age-related increases in adiponectin and inflammatory markers are unrelated [26], which supports our finding.

Several studies have also shown that plasma adiponectin levels are high and indicate a poor prognosis in some critical conditions such as severe CVDs [16], [17]. Accumulation of adiponectin in patients with diseases such as heart failure or chronic kidney disease may reflect the emaciation and malnutrition that characterize these diseases, and therefore, such an accumulation is a marker of poor prognosis [27]. However, the nutritional status, including albumin levels, hemoglobin levels, and BMI, was similar among the three subgroups with different levels of frailty severity in this study. Thus, malnutrition is not the only factor affecting plasma adiponectin levels in elders.

Aging is associated with weight loss and loss of skeletal muscle mass and strength (sarcopenia) [28], which are prominent predictors of mortality in the elderly population [29]. Body weight is an important factor in the regulation of adiponectin expression. Plasma adiponectin levels decreased in obese patients [6] and increased after weight reduction [30]. It has been suggested that high adiponectin levels in elderly persons may be a consequence of weight loss and sarcopenia. Even though the weight and BMI values were not different among the different subgroups in this study, the sample size in this study is not enough to exclude the effect of weight.

Age-related decline in renal function might cause a reduction in renal adiponectin clearance that may contribute to the increase in adiponectin levels in the elderly [24]. However, renal function, which was estimated using modification of diet in renal disease (MDRD)-simplify-GFR, was similar among the different subgroups in this study.

Thus, sex is an important factor not only affecting plasma adiponectin levels [31], but also their relations to frailty severity. The finding suggests that adiponectin may have different regulation in frail male and female elders. This may not relate to the adiponectin gene that explains 6.7% of the phenotypic variation because the gene is unlikely to cause sex differences in adiponectin levels [32]. A potential explanation could be sex hormone levels. Sex-hormone modulation of adiponectin production may provide protection from adiponectin elevation with frailty in the female elders.

In addition to sex, nutritional status and body weight, other factors affecting plasma adiponectin levels include menopausal status, and medications such as β-blockers and angiotensin II receptor blockers [33], [34]. However, the age of the study subjects was greater than 65 years, and therefore, all the female participants were menopausal. Furthermore, the use of β-blockers and angiotensin II receptor blockers was not different among the three subgroups.

Factors such as malnutrition, weight loss, comorbid diseases, and medication use did not explain the high levels of plasma adiponectin in frail elders. Geriatric frailty, which causes disturbances in energy homeostasis, is associated with sarcopenic obesity and physical inactivity [35]. Thus, the elevation of plasma adiponectin levels in the frail elders may be due to age-related homeostatic dysregulation [18]. Further investigation is warranted to define the underlying mechanisms.

The small sample size is a limitation in this study, which limits the number of covariates that can be added to regression models and is underpowered in detecting a significant interaction by sex. A larger-scale study to confirm the results obtained in the small-scale pilot study is necessary. On the other hand, circulating adiponectin has three isoforms: high-, medium-, and low-molecular-weight adiponectin [36]. Previous studies have shown that high-molecular-weight adiponectin exerts a protective role as an antidiabetic and anti-atherogenic hormone [36], [37]. However, a recent study showed that low-molecular-weight adiponectin has a protective role in aging [38]. Therefore, further studies on the adiponectin isoforms in elders are necessary to elucidate the role of adiponectin in geriatric frailty.

In conclusion, plasma adiponectin levels correlate positively with an increasing number of components of frailty especially in male elders. The difference between the sexes suggests that certain sex-specific mechanisms may exist to affect the association between adiponectin levels and frailty. Further study is required to elucidate the underlying mechanisms.
